# Accidental Falls in Patients with Hyperkinetic Movement Disorders: A Systematic Review

**DOI:** 10.5334/tohm.709

**Published:** 2022-10-07

**Authors:** Carl N. Homann, Barbara Homann, Gerd Ivanic, Tadea Urbanic-Purkart

**Affiliations:** 1 Department of Neurology, Medical University Graz, Graz, Austria; 2 St. Elizabeth University of Health and Social Work, Bratislava, Slovakia; 3 Institute for Orthopedic and Cardiological Rehabilitation, Privatklinik Ragnitz, Graz, Austria

**Keywords:** accidental falls, fractures, injuries, accident prevention, hyperkinetic movement disorders, basal ganglia disorders, essential tremor, restless legs syndrome, Huntington’s chorea, dystonia, myoclonus, tic disorders, ataxia, athetosis, stereotypies, ballism

## Abstract

**Background:**

The significance of falls and their repercussions in Parkinson’s disease has been extensively researched. However, despite potentially serious effects on health and quality of life and negative impact on the healthcare system, there is not a sufficient understanding of the role of falls in hyperkinetic movement disorders (HKMDs). This review aims to provide an overview of the prevalence of falls, injuries, and preventive measures in the most common HKMDs.

**Methods:**

Studies up to May 1, 2022 were searched in PubMed using Medical Subjects Headings of relatively prevalent HKMDs associated with the terms “accidental falls”, “injuries”, “fractures”, and “accident prevention”.

**Results:**

In our review of 37 studies out of 155, we found evidence that for several HKMDs, such as spinocerebellar ataxia, essential tremor, Huntington’s disease, and dystonia, fall risk is increased. Falls were reported in up to 84% of spinocerebellar ataxia patients, 59% of essential tremor patients, and 79% of Huntington’s patients, with 65% of the latter falling frequently. Injuries occurred in up to 73% in Huntington and 74% in ataxia patients. Most of the common diseases characterized by HKMDs were investigated for both fall causes and consequences, but prevention studies were limited to spinocerebellar ataxia and Huntington’s disease.

**Discussion:**

The limited available data suggest that patients with several HKMDs can be considered to be at increased risk of falling and that the consequences can be serious. As a result, physicians should be advised to include fall exploration in their routine workup and provide advice for safer mobility. In general, more research into fall-related concerns in HKMDs is necessary.

**Highlights:**

In contrast to Parkinson’s disease, the prevalence of accidental falls, their repercussions, and preventive strategies are under-investigated in hyperkinetic movement disorders (HKMDs). Several HKMDs such as essential tremor, ataxia, and Huntington’s disease have reported fall rates of up to 84% and fall-related injury rates of up to 74%. Therefore, routine examinations of HKMD patients should include a fall exploration and provide advice on safe mobility.

## Introduction

Traditionally, movement disorders have been attributed to a dysfunction of basal ganglia-related circuits resulting in poverty or excess of movement. In recent years, researchers have established that brain regions previously thought to be remote from the basal ganglia (BG), for example, the cerebellum and the pedunculopontine nucleus, also play an essential role in these disorders [1]. Chorea, tremor, dystonia, tics, myoclonus, ataxia, and symptoms of restless legs are the most frequent hyperkinetic movements, whereas akinetic-rigid symptoms of Parkinson›s disease (PD) are the most prevalent hypokinetic movement dysfunctions. Genetically and clinically, movement disorders comprise a group of conditions that are heterogeneous and some are progressive neurodegenerative diseases, while others are less dynamic, non-degenerative conditions. While gait and balance difficulties have been extensively studied in some of the common movement disorders, such as PD, Huntington’s disease (HD), and the spinocerebellar ataxias (SCAs), falls have received less attention in others where they are more subtle. This is despite their potentially serious consequences, with falls and injuries being the most concerning [2].

The World Health Organization defines falls as an event that results in a person coming to rest inadvertently on the ground floor or other lower level [3]. The corresponding Medical Subject Headings (*MeSH*) term “accidental falls” was introduced in 1987, and here falls are described as an incident that occurs due to slipping or tripping and may result in injury [4,5]. *MeSH* is the National Library of Medicine’s (NLM) controlled vocabulary thesaurus for indexing every article in *PubMed*. Given that all MeSH terms are pre-defined and have synonyms included, they are deemed effective in searching for meaning rather than only looking for words appearing in the text or the abstract.

In biomechanical terms, falling is caused by a loss of balance control, which refers to maintaining the body’s center of mass (COM) in a horizontal plane within the boundaries of the base of support (BOS) [6]. When the vertical projection of the COM moves beyond the BOS, then without a successful balance-correcting response to arrest the falling state and regain postural equilibrium, the incident is inevitable. In BG disorders, both the ability to maintain the COM and carry out corrective measures may be affected. Sensory signal processing and integration are critical for this, and the BG is a pivotal contributor to the underlying multisensory network. According to studies employing objective posturography data, various movement disorders, including PD, HD [7], cervical dystonia (CD) [8], essential tremor (ET) [9], restless legs syndrome (RLS) [10], Tic disorders (TDs) [11], and several of the SCAs [12], have postural control impairments indicative of an increased risk of falling. The most frequent biometric findings, which are regarded responsible for this, were increased truncal sway amplitude and velocity. Another common finding across the various movement disorders was that the more complex the task, such as the high demand for multimodal sensory integration, the more difficult it was for patients to restore their COM to the center of the BOS [11,12]. An individual’s ability to maintain balance can be further hampered by various biological, behavioral, environmental, and socioeconomic factors. Many of these risk factors and how they contribute to falls have been extensively studied in hypokinetic movement disorders, such as PD [13], but there is much less information on their effects on hyperkinetic movement disorders (HKMDs).

Even in the general population, falls are a common, serious, growing public health issue and driver of medical costs [13]. The elderly are particularly at risk. Approximately 30% of adults over 65 fall each year [14] with fatal and non-fatal fall injury rates of 17%, and 31%, respectively [15]. In survivors, falls often lead to decreases in mobility and loss of independence [14] and thus have a substantial negative impact on social and psychological well-being [16]. Patients with neurological diseases tend to fall more often and suffer more severe consequences [17]. In a study of older adults living in the community, the prevalence of falls was three times higher in neurological patients and eight times higher in Parkinson‘s patients than in healthy controls. Also, these patients were found to have worse outcomes [17,18,19,77]. In contrast to Parkinsonian syndromes, only a limited amount of data on falls and related issues is available for HKMDs. This study aims to present an overview of falls, their prevalence, causes, repercussions, and prevention for each of the most common diseases characterized by HKMDs. This might not only provide researchers with more insight into areas that require further research but it can also be used by physicians to determine where there is sufficient evidence to adjust their treatment strategies. For example, it may lead to a more liberal or more restrictive approach when prescribing certain medications or suggesting exercise programs and walking aids.

## Methods

The study search was conducted through PubMed for all English, French, Spanish, and German articles until May 1, 2022. As part of a standard search protocol, the following keywords or Medical Related Subjects (MeSH) were used to specify the literature search: The term “accidental falls” was cross-referenced with the terms “restless legs syndrome”, “huntington disease”, “dystonic disorders”, “essential tremor”, “myoclonus”, “athetosis”, “tic disorders”, “stereotypies”, “ballism”, and “ataxia”.

In addition, a link was made to each of these terms with “wounds and injuries” or “fractures, bone” and “accident prevention”. In other words, we extracted from the selected articles on falls and HKMDs data about the ratio of falls, causes of falls, sequels produced by falls, and preventive actions.

Furthermore, the bibliographies of the identified publications were reviewed for further appropriate studies. By comparing author names, interventions, publication dates, sample sizes, and outcomes, dual publications were detected and subsequently excluded. To better understand the possible impact of the included studies, they were graded by the quality of evidence and favorability of outcome.

According to predetermined standards, the degrees of evidence were graded from A to D (Table 1). If there were one or more meta-analyses or systematic reviews, they were placed in category A; if there were several randomized controlled trials (RCTs) or representative cohort studies (RCSs), they were placed in category B; if there was one randomized controlled trial (RCT) or one representative cohort study (RCS), they were placed in category C; and if there were just observational studies, they were placed in category D.

**Table 1:** Quality and outcome of studies on fall prevalence, causes, consequences and prevention in patients with hyperkinetic movement disorders.


DISORDER	PREVALENCE	CAUSES	CONSEQUENCES	PREVENTION

**Huntington’s disease**	C ++	D ++	D ++	A –

**Restless Legs Syndrome**	D (+)	D (+)	D (+)	ᴓ

**Tic disorders**	C (+)	C (+)	D +	ᴓ

**Essential tremor**	D ++	D ++	ᴓ	ᴓ

**Dystonia (cervical)**	D ++	D +	D +	ᴓ

**Spinocerebellar Ataxias**	D ++	D +	D +	B +

**Myoclonus**	ᴓ	D (+)	ᴓ	ᴓ

**Ballism**	ᴓ	ᴓ	ᴓ	ᴓ

**Stereotypies**	ᴓ	ᴓ	ᴓ	ᴓ

**Akathisia**	ᴓ	ᴓ	ᴓ	ᴓ


**Quality:** D: observational studies only, C: one randomized controlled trial (RCT) or one representative cohort study (RCS), B: more than one RCT or RCS, A: one or more meta-analysis or systematic review. **Study-outcome:** Favorable: +, very favorable: ++, unfavorable: –, data only allows an indirect assumption: (+). ᴓ: no studies.

Positive results regarding falls were rated as favorable (+) and negative results as unfavorable (–), with the degree of favorability further broken down into very favorable (++) in the case of multiple positive investigations and very unfavorable (––) in the case of multiple negative investigations. The data was rated as potentially favorable (+) if it permitted an indirect inference.

## Results

### Article selection

We retrieved 155 studies combining HKMDs with falls when screened by titles and abstracts through the above mentioned systematic search strategy. These 155 studies consisted of six articles for essential tremor, three for restless legs syndrome, 15 for Huntington’s disease, 25 for dystonia, 17 for myoclonus, 87 for ataxia, and two for tic disorders. There were no articles for athetosis, stereotypies or ballism. Through full-text reading, we excluded 122 studies not meeting the criteria (Language, non-human studies, or no quantitative information on the topic). We removed one revoked and one duplicate publication but added six found in bibliographies, leaving us with 37 scientific articles to use for this review. (Supplementary Figure 1).

### Huntington’s Disease

***Fall rates:*** The risk of falls in Huntington’s disease (HD) patients was examined in seven studies. (Table 2) Busse et al. detected a 79.2% one-year fall rate in his investigation of 24 HD patients. Out of these, three quarters fell more often than once [20]. Grimbergen et al. conducted a partly retrospective and partly prospective study in which 60% of 45 moderately impaired HD patients reported falling two or more times in the preceding year, and 40% reported falling at least once during the prospective follow-up period of three months [21]. In the study by Chel et al., 15 of 28 (54%) HD patients were classified as recurrent fallers, defined by having two or more falls per year [22]. Prucel et al. report in their cross-sectional investigation of 17 HD patients a fall rate of 58,8%, and an average number of self-reported falls over the previous twelve months that was considerably greater than in 17 healthy participants [23].

**Table 2:** Summary of content of studies on fall prevalence, causes, and complications in patients with hyperkinetic movement disorders.


DISORDER	FALL RATES	CAUSES	COMPLICATIONS

**Huntington’s disease**	60–79.2% (1yr)	Environmental hazards; involuntary movements, disturbance of gait and posture; multitask situations	Physical harm (including injuries and death), psychological impairment, and social status deterioration

**Restless Legs Syndrome**	ᴓ	Anecdotal: hypersomnolence, cognitive impairment, drug-intake, orthostatic hypotension	Anecdotal: physical harm, including fractures

**Tic disorders**	ᴓ	Abnormal gait and balance patterns	Physical harm, including fractures and traumatic brain injury

**Essential tremor**	50.7–58.7% (1yr)	Abnormal gait patterns	ᴓ

**Dystonia (cervical)**	27.3–39% (6 m)	Anecdotal: Environmental	Psychological consequences

**Spinocerebellar Ataxia**	73.6–84.1% (1yr)	Disease severity, age, and SCA type	Physical harm and social status deterioration

**Myoclonus**	ᴓ	ᴓ	ᴓ

**Ballism**	ᴓ	ᴓ	ᴓ

**Stereotypies**	ᴓ	ᴓ	ᴓ

**Akathisia**	ᴓ	ᴓ	ᴓ


Fall rates: (1yr) and (6 m) meaning annual and 6-monthly incidence rates.

A recent study by Terroba-Chambi et al. revealed that seven (35%) of 20 HD patients with falls reported just one fall in the last 12 months, while 13 (65%) reported two or more falls [24] Kalkers et al. reported a 28.8% fall rate in 158 institutionalized HD patients during the prior 30 days [25].

In the most extensive research conducted so far, Zarowitz searched data extracts of 249,811 residents derived from prescription claims records collected from about 19,000 long-term care institutions in 48 states and Canada. They aimed to investigate the clinical features of nursing home residents with HD. One hundred and sixty-two of the 340 HD patients (47.6%) had at least one fall over the 18-month reporting period [26]. It is worth mentioning that fall propensity varies considerably throughout the disease course, and that in later stages when patients become wheelchair-bound or bedridden falls become less frequent.

***Predisposing conditions:*** Extrinsic factors precipitating falls were investigated by Grimbergen et al. [21] Of the 40 participants who submitted questionnaires on fall specifics, 37 (92.5%) stated they fell in a familiar environment, and 23 (57.5%) said they fell inside. Self-reported causes were in 10 cases (25%) an obstacle on the floor, in seven (17.5%) a slippery or uneven surface, and in eight (20%) the climbing of stairs.

The intrinsic causes of falls in HD often originate from multiple sources, but can generally be classified as motor and non-motor alterations. Motor symptoms so far investigated in HD-fallers include involuntary movements and disturbance of gait and posture [21]. Grimbergen et al. found correlations of falls with metric scores of choreatic trunk movements, brady- and hypokinesia, and balance impairment.

The non-motor impairments most often blamed for contributing to falls in HD consists of cognitive decline, reduced wakefulness, and emotional and behavioral alterations [21,27]. The fact that falls were most common in so-called “multiple task” situations (35.0%), which are the most difficult for people with cognitive impairment to handle, underlines the importance of an unimpaired mental state for safe posture and movement. Studies on HD patients have shown that cognitive tasks interfere with walking and balance under dual task-settings, and that these multi-tasking difficulties are related to the extent of striatal degeneration in HD brains [28]. Furthermore, the risk of falling during dual-tasks is known to be linked to the degree of cognitive impairment in older persons [29]. It appears that walking relies on numerous cognitive abilities, including executive-attentional function, visuospatial ability, and memory. In normal situations this uses up most of the capacities [29]. When cognitive resources are few, they are readily overstretched leading to errors in the synchronization of movement components and thereby to stumbles and falls.

Other non-motor difficulties preceding a fall, such as dizziness, were documented by Grimbergen et al. in 7.5% of his patients.

***Consequences and complications:*** The fall consequences so far examined were physical harm, including injuries and death, psychological impairment, and social status deterioration. In the small cohort of patients in the study by Grimbergen et al., injury rates were as high as 72.5%. However, presumably, because individuals mostly fell indoors, none of these were severe injuries.

Even so, while there are no further systematic investigations on this subject, data suggest that falls in HD can cause more than just minor injuries. In the study by Zarowitz et al., on clinical features of nursing home residents with HD derived from the extensive database of prescription claims in North America, 66 of 162 fallers (41%) suffered any injury as a result, while as many as 14 (9%) had a major injury [26]. Ground level falls with head trauma are generally one of the leading causes of subdural hematoma (SDH) [30,31]. There are two reports on fall-related SDHs in HD patients. For example, a SDH occurred in one patient in the active drug arm of a small clinical trial of a compound proposed to improve cognitive functioning [32].

Pechlivanis et al. investigated the prevalence of chronic SDHs in HD patients seeking hospital treatment [33]. During the one-year research period, they treated 58 HD patients with this condition in their neurosurgery department, four of whom (6.9%) required surgical evacuation.

However, we must equally suppose that some falls are serious enough to be fatal. In a population-based study using the Norwegian Cause of Death Registry, Solberg et al. studied under which circumstances HD patients died and compared them with data of the general public [34]. In 1986–2015, they identified 559 deceased persons with HD. Eighteen of those (3.2%) died because of “other injuries”, which the authors attributed mainly to accidental falls.

Unlike physical consequences of falls, psychological consequences have been less studied. According to Grimbergen et al., fear of falling, usually a frequent and severe complication, was present in only 15% of HD patients [21]. The lack of insight among HD patients may explain this surprising finding, which has not been formally investigated in detail.

Finally, the social ramifications of falls and related injuries in HD can also be quite severe. Falls in people with HD are assumed to frequently result in the loss of autonomy and transfer to nursing homes [35], further impairing their already low quality of life. This has an impact on regional and national health budgets as well. Future research should include these elements, we believe.

***Preventive action:*** Based on the findings of the systematic review by Quinn et al., including 23 quantitative studies and three qualitative studies [36], aerobic exercise, alone or combined with resistance training, improves motor function and fitness, while supervised gait training enhances spatiotemporal characteristics of gait. Nevertheless, they could not show that physical training significantly reduced fall rates, despite weak evidence for improving balance.

### Restless Legs Syndrome

***Fall rates:*** Scanty scientific data from three case reports seem to indicate that RLS patients can tend to fall. However, while there would be a need for it, no systematic studies have investigated fall prevalence or accident causes in RLS patients.

The paper by Kuzniar et al. reports a 73-year-old woman with uncontrolled RLS resulting in multiple falls [37]. Throughout the day, she fell several times, while most of the night, her RLS symptoms kept her standing or walking. In another case report, Krøigård et al. reported repeated nocturnal falls in a 75-year-old man with severe RLS and persistent periodic limb movements throughout awake and sleep [38]. Becerra et al. report a 71-year-old RLS patient with a history of medication-induced orthostatic hypotension who had five falls in less than eight months [39].

***Predisposing conditions:*** Several mechanisms have been have been proposed to explain RLS patients’ falls: Cumulative sleep deficit, resulting in daytime hypersomnolence, difficulties concentrating, and delayed reflexes, as cited by Kuzniar et al. and Krøigård et al. [37,38] Kuzniar et al. also identified cognitive impairment, to be linked to gait slowness and instability [37]. They and Becerra et al. also refer to drug-induced daytime drowsiness as seen with dopamine agonists [37,39] or antidepressants [37]. Finally, as highlighted by Becerra, ropinirole induced orthostatic hypotension can also be a risk factor [39].

***Consequences and complications:*** There are no systematic investigations on the consequences of falls, particularly not on fracture or injury prevalence in patients with RLS. However, in the case report mentioned above by Kuzniar et al., the authors describe an increased fracture risk after falls on top of a high fall propensity [37]. They state that due to her multiple falls, the patient sustained, on several separate occasions, fractures of both forearms, ribs, and nose. Becerra et al. describe that two (40%) of their patient’s falls required hospitalization but do not give details about the sustained injuries [39]. Unfortunately, neither of the authors provided data on bone mineralization or vitamin D levels frequently found diminished in RLS [40,41], which could have been a possible explanation for the seriousness of the injuries [42,43,44].

### Tic disorders

***Fall rates:*** As for the prevalence of falls among tic patients, there are only sparse data, including one investigation from a specialized outpatient clinic and two systematic population-based studies on traumatic injuries, both from the same extensive health care database. However, investigations are limited to patients with Gilles de la Tourette syndrome (GTS) and only allow indirect assumptions about tic patients’ increased vulnerability to falling and related injuries.

Cheung et al., when searching the outpatient data of their movement disorder clinic to investigate clinical features of patients with malignant GTS, found that 17 (5.1%) of 333 patients with tic disorders met the criteria for malignant GTS [45]. After a series of severe falls, one of these, a 43-year-old woman with malignant GTS, first diagnosed at 11 years of age, lacerated her scalp. She also had several other injuries like a C3–4 cervical disc herniation due to a head jerk tic, an elbow fracture, and an ovary and bladder damage due to self-inflicting behavior [45]. Lu et al. used claims data from 1 million Taiwanese National-Health Insurance program members to analyze the risk of fractures in children with GTS. The 1,258 children diagnosed with GTS had a 1.27-fold greater fracture rate than the control group, which was matched for sex, age, residence, and parental profession [46]. Chen et al. evaluated whether GTS patients were associated with an elevated risk of traumatic brain injury using the same data set as Lu but included a larger population by extending the research period. They found a 1.59-fold greater risk in children with GTS than in non-GTS controls [47]. The nature of the data made it impossible to identify how many of these injuries were actually caused by accidental falls.

***Predisposing conditions:*** There are also no systematic studies on the causes of falls in tic disorders. However, data of some relatively small studies suggest abnormal gait and balance patterns in GTS patients [48,49], which could potentially increase the risk of falls in other HKMD populations [21]. Contrary to findings in other conditions [50], both the investigation by Chen et al. and by Lu et al. indicated that in GTS patients, antipsychotic use was associated with a lower rather than a higher risk of injuries [46,47]. Until further research is done in this area, our best guess is that in GTS, the net effect of antipsychotic agents could be fall-reduction through suppression of erratic behavior, outweighing their fall provocation through sedation.

***Consequences and complications:*** There are also no systematic investigations on the consequences of falls in patients with simple tics or GTS. The patient in the aforementioned case report by Cheung et al. had many falls in addition to various injuries, some of which were severe and included skull fractures [45]. The investigations by Chen et al. and Lu et al. revealed that tic patients are more prone to fractures. This, as reported in other HKMDs, might be explained by dietary or drug-induced bone structure alterations [51,52].

***Preventive actions:*** There are no investigations on physiotherapy or other interventions to mitigate fall risk in tic disorders.

### Essential tremor

***Fall rates:*** Two studies investigated fall risk in patients with essential tremor (ET). Rao et al. found that 132 people with ET have more frequent falls than 48 healthy controls with fall rates of 54.5% and 31.2%, respectively [53]. In a recent publication, Louis et al. documented in 199 ET patients an average one-year fall rate of 1.2 [54]. He stressed that the fall risk in individual ET patients varies and can be moderate to high, ranging from 0 to 24 falls. This basically confirms findings of a previous investigation of the same group [55].

***Predisposing conditions:*** Several possible intrinsic causes for falls in ET patients, some psychological and some motor function-related, were documented. Rao et al. and Louis et al. both focused on the influence of cognitive dysfunction. According to data by Rao et al., 58.7% of the 63 patients with poorer cognitive test scores fell, which was significantly higher than the 50.7% fallers among the 69 patients with better cognitive test scores [53]. In line with these findings, Louis et al. discovered that patients with weaker global (p < 0.05) and functional cognition (p < 0.05) had a higher number of falls [54].

Common motor factors for frequent falls are gait and balance problems, as detailed in a case report by Cheung [45]. Several systematic studies have shown that in ET, balance and gait disturbances often occur together [56]. Research by Rao et al. indicated that changes in velocity, cadence, step length, double support, step time difference, stride length, and stride time were all associated with an increased prevalence of falls [53]. Baudendistel et al., investigating turning behavior and falls in 15 ET patients and 15 PD patients over the three-month study period, found that increasing turning cadence and decreasing forward cadence were significantly associated with higher fall risk [57].

***Consequences and complications:*** In the literature search, we did not find any systematic studies for fall-related injuries or fracture frequency in patients with ET. There are also no studies on altered bone health as a potential risk factor for fall-related injuries.

***Preventive actions:*** No studies on preventive measures are available.

### Dystonia

***Fall rates:*** Data on fall incidence for patients suffering from dystonia are somewhat limited. In a study investigating the one-year fall incidence of 548 neurological inpatients, one out of the two patients admitted with dystonia reported to have had a fall [58]. Boyes et al. showed in a more extensive study an increased risk of falling in patients with focal dystonia [59]. These patients all had cervical dystonia (CD), i.e., a location where an elevated fall risk would not have been expected from the outset. In this examination of 122 subjects diagnosed with CD, an increased 6-month incidence rate of 39% was described [59]. Five of twenty-two CD patients (27.3%) in a prospective study undertaken by the same authors reported falling during the 6-month follow-up period [60]. It is worth mentioning that no studies examining fall rates in patients with other focal dystonias or with generalized dystonia have been performed.

***Predisposing conditions:*** Barr et al. undertook a small research study on 10 CD patients to evaluate the causes of this increased fall incidence. They observed that CD patients exhibited motor abnormalities in balance, gait, and stepping responses [61]. Pathophysiologically, postural control and gait impairment were described as a result of decreased sensorimotor-control processes produced by altered muscle afferent input from a protracted twisted neck position [61]. Furthermore, fear of falling, a well-known psychological risk factor for future falls, which can be both a cause and a result of falls, was more prevalent in dystonia patients than in normal controls [61]. Boyce et al. described the circumstances that presumably led to the fall, mainly also indicating a link to balance deficits [62]. Three of the five individuals experienced long-term dizziness, which they thought contributed to their falls. One person described stumbling over a rock while going backward on a beach, while the other tripped while standing on a stool reaching for an object on a high shelf.

***Consequences and complications:*** As with other HKMDs, evidence is scarce on fall-related consequences in dystonia. There is one small prospective study on falls exploring physical repercussions. However, none of the five CD participants in the investigation by Boyce et al. who reported falling in the preceding six months sustained severe injuries, and none sought medical assistance at the time of the accident [62].

As indicated before, one of the most frequent and consequential fall-related psychological sequelae is fear of falling. In the research study conducted by Boyce et al., fear of falling was observed to be high and balance confidence low in their 122 participants with CD, and both were worse in those who had previously fallen [59]. In their small above cited research, Barr et al. confirmed that his 10 CD patients expressed higher fear of falling and lower fall self-efficacy than ten healthy control subjects did. Nevertheless, they did not investigate whether and how often this occurred due to a previous fall [61].

***Preventive actions:*** There is no research on means to prevent falls in patients with dystonia.

### Spinocerebellar Ataxia and other Ataxias

***Fall rates:*** Few studies demonstrated that patients with spinocerebellar ataxias (SCA) have significant static and dynamic balance impairment and a higher risk of falls [63,64]. In a study of the EuroSCA Fall Study project directed by Fonteyn et al., 73.6% of 228 SCA patients fell at least once during the one-year study period [63]. In prospective research conducted by the same group of investigators, 84.1% of 113 SCA patients who recorded their falls in a diary over a year had fallen [65].

***Predisposing conditions:*** Morton et al. investigated the causes of balance and functional disability and revealed that they were influenced mainly by disease severity, age, and SCA type [66].

***Consequences and complications:*** The EuroSCA Fall Study project investigated the physical consequences of falls and discovered that 74% of the 166 fallers were affected by fall-related injuries [63]. Morton et al. focusing on social repercussions, found that falls had a detrimental effect on the patients’ ability to function, in particular concerning self-care, transfers, and locomotion [66].

Two historical case reports reported fractures following spontaneous falls in patients with ataxias; one concerned the hip [67], and the other both tibia and fibula [68]. There are few systematic investigations on bone health, including one small study on Friedreich’s ataxia [69] and a case-control study on patients with spinocerebellar degeneration [70]. These two studies showed significantly reduced bone mineral density and vitamin D serum levels compared to healthy controls. Nonetheless, a direct link between bone structure changes and fall-related injuries was not examined, which calls for further research in this area. This we deem particularly important as low vitamin D levels, which is a commonly but often overlooked occurrence in individuals with HKMDs, need to be corrected to prevent bone demineralization and fractures due to falls [40,41].

***Prevention:*** There are two clinical trials on the effectiveness of rehabilitation interventions in cerebellar ataxias. Ilg et al. performed a prospective neurorehabilitation study on 15 individuals with hereditary cerebellar ataxias, three of whom had SCAs [71]. They discovered that four weeks of intense coordination training with three one-hour weekly sessions dramatically lowered known markers of falling risk such as the Berg Balance Scale (BBS) scores and improved motor performance and ataxia symptoms. Some benefits remained up to a year after the treatment sessions had ended [72]. The complete physiotherapy program comprised exercises to enhance static and dynamic balance (such as standing on one leg and ascending stairs), whole-body motions, fall prevention measures, and falling tactics. Similarly, Santo de Oliveira et al. found that a slightly modified and decreased program, done twice weekly for four weeks, improved balance and reduced the risk of falling in 11 individuals with SCA [73]. It is, however, impossible to draw a direct inference from these relatively small studies because none of them used falls directly as an outcome measure.

### Other Hyperkinetic Movement Disorders

No research has been conducted on the prevalence, causes, or preventative measures of the various other disorders characterized by abnormal involuntary movements, such as myoclonus, stereotypies, akathisia, athetosis, and ballism. Neither are there investigations on bone mineralization, fractures, or fall prevention in these conditions.

## Discussion

This is the first systematic study that reviews the role of falls and related issues in HKMDs. Given that HKMDs are a set of disorders that are not uncommon, and falls often have severe consequences, this is remarkable. In summary, we found a growing body of research suggesting that HKMDs in their entirety are a group of conditions with a high propensity for falls. This is no surprise, considering the basal ganglia and other structures involved in movement disorders like the cerebellum are critical for ensuring posture and gait stability when intact [2]. Overall, the level of evidence for the investigated aspects, however, differs widely, and in general, it is not very high. Some quality data are available for HD, ET, and RLS, whereas only a few and incomplete investigations exist for dystonia, myoclonus, and SCA. No studies at all have been found on stereotypies, akathisia, athetosis, and ballism (Table 1).

### Fall risk

We reviewed several studies that reported a high incidence of falls in patients with specific HKMDs, providing incidence figures for HD patients from 60 to 79.2% [20,21], ET patients from 50.7 to 58.7%, and SCA patients from 73.6% to 84.1% [77]. However these findings may not represent the whole group, as all SCA types cannot be viewed as equal, and there is such a wide variety of phenotypes, it may be necessary to investigate each type separately.

HKMDs patients also tend to fall repeatedly. In the investigations mentioned above, 58.3% of HD patients reported recurrent falls. For other HKMDs such as RLS [37] and tic disorders [45], there are but single case reports of repeated falls.

Compared to that, there are multiple investigations on PD and falls, including four recent meta-analyses, which drew their data from between 17 to 88 studies. Prospective studies show that between 45% and 68% of PD patients fall each year [74], with 50–86% falling recurrently [74]. Elevated fall risk is also a common finding in other neurological conditions. A prospective study on community-dwelling individuals showed that the one-year incidence for neurological patients was three times higher than that of age-matched controls [74]. Out of 228 consecutive physically independent community dwelling patients aged 60+ years who were treated in a general neurological outpatient clinic without referral requirements, 46.5% fell, with several biological factors contributing to fall risk, including multiple neurological comorbidities, lower Activities-Specific Balance Confidence scores, and depressive symptoms. In this study, a higher risk than in PD was only seen in stroke with 89%. Other neurological diseases with a high rate of falls were dementia with 60%, epilepsy with 57%, and polyneuropathy with 55%. These results align with what we saw in SCA, HD, and ET, indicating they too fall into the high-risk category.

### Predisposing conditions for falls

Although there are several studies on gait and balance deficits in various HKMDs, a direct association with fall risk was only examined in HD, ET, GTS, and SCA. These studies were not very large and the correlation between HD and falls was rather modest. This is in contrast to the general public, where gait and balance deficits seem to carry the most weight among all fall precipitating factors with an OR of 2.13 [75,79]. An altered mental state, was yet another predisposing condition identified for causing frequent falls in HD and ET [21,27]. Drowsiness, as a side effect of medication, was described in cases of falls of RLS patients [37,39]. However, otherwise, research on medication as a risk factor for falls has surprisingly received little attention in HKMD research.

A significant point to be noted is that most research has focused on the causal relationships between falls and general biological risk factors such as aging, disease severity, cognitive impairment, and attention deficit rather than on specific factors pertaining to hyperkinetic movements themselves. Only in HD we found information correlating metric scores of choreatic trunk movements with fall propensity [21]. Research into these specific factors and specifically how hyperkinetic movements contribute to fall rates would be useful, as it would impact clinical practice and research.

While there is some information on biological risk factors for falls in HKMDs, there is limited data on behavioral risk factors such as hazardous decision-making and alterations in emotional behavior reported in HD [21,27]. Furthermore, environmental fall risk factors such as obstacles on the floor and slick, uneven surfaces, which are also investigated primarily in HD, are under-represented in the literature [22]. In contrast, socioeconomic fall risk factors are not addressed at all in HKMD research, suggesting that these might be interesting future study areas.

The research situation is much better for other neurological diseases. For instance, in PD, just as described before in the fall propensity section, research on predisposing factors is abundant, including several meta-analyses and systemic reviews. There, aside from impaired gait and balance and cognitive deficits, the most consistently mentioned factors include a history of past falls, disease severity, reduced mobility, and lower limb muscle strength [76,80].

### Consequences and complications of falls

There are but two systematic studies, both for HD patients, describing higher fall-related fracture rates in HD than in controls [78,81,82] For RLS [37] and tic disorders [45] there are only case reports. Injury rates were provided for HD from 41% to 73% and for ataxia at 74%. Whereas in most HKMDs the injuries were primarily mild, in HD 14% of the injuries were severe.

Psychological and social consequences of falls studied in HD included fear of falling [21]. poor quality of life [21,45], reduced activity levels [21], as well as loss of autonomy and transfer to nursing homes [35]. In GTS, quality of life was negatively impacted, while in ataxia, it had a more pronounced impact on the ability to function, particularly in self-care, transfers, and locomotion [66]. There are no data at all on the economic consequences of falls in HKMDs.

As for fall repercussions, the data situation for PD is considerably better than that for HKMDs. There are at least five investigations on fall-related injuries in PD patients. This research suggests that these injuries occur in up to 50% of falls [83] and constitute one of the top causes of increased health services utilization and overall costs [84]. Similar data exist for other neurological diseases [17].

For obvious reasons, further research on fall repercussions in HKMDs is needed, suggesting that this would be another worthwhile future project.

### Preventive actions

Summarizing the current research on neuro-rehabilitation as a measure of fall prevention, we must state that it is deficient for the majority of HKMDs and, when it is present, of poor quality. Only in SCA [77] and HD [36] was fall prevention specifically researched. In the former, outcomes relating to reducing falls were indirectly positive, in the latter negative. Although there are multiple lines of evidence that show high-intensity, customized physical rehabilitation programs, particularly for gait and balance training, can enhance motor function, the impact on lowering falls has seldom been studied. The studies Ilg et al. [71] and by Santo de Oliveira et al. [73] provide some information on the efficacy of rehabilitation therapies on the risk for unintentional falls, although they did not primarily monitor fall frequency but only indirect indicators for fall risk. There is a similar pattern in neuro-rehabilitation research for HD patients: Although HD patients consistently appeared to benefit from intensive rehabilitation in terms of improving function, mobility, ataxia, and balance, the number of falls was not reported as an outcome measure in most of the studies. Only the research by Quinn et al. [21] looked into fall prevention but could not demonstrate that physical training significantly decreased fall rates.

For individuals with other neurological disorders, on the other hand, there is plenty of evidence available. A total of 15 studies, six systematic reviews and nine meta-analyses, include systematic investigations of exercise interventions [85]. The data of these surveys aggregated suggest that exercise interventions reduce the number, frequency, and rate of falls among people with neurological disorders, including cognitive impairment, dementia, and PD [85]. There has been plenty of research done just on PD. O’Malley et al., for example, in a recent compilation of systematic reviews on the effectiveness of exercise-based interventions at reducing falls, identified 11 PD studies of poor to intermediate quality of evidence. The majority of evaluations found the intervention to have a significant effect on documented fall outcomes, but three found inconsistent results on both efficacy and outcomes [86]. We conclude that even though neurorehabilitation in movement disorders has made progress due to better understanding of pathophysiology and compensatory mechanisms, the rise of promising approaches, such as increased practice intensity, and the use of new technology, high quality research continues to be needed both in areas where data are abundant and in areas where they are scarce.

### Strengths and limitations

We also faced several limitations in our research. First and most importantly, like most other reviews dealing with falls, we have the problem that the number of falls is likely to be under-reported. A majority of the included papers used self-reported recall assessments that are suspected to be tainted by intentional omissions due to patients’ fear that their mobility will be restricted or unintentional errors due to memory deficits. Daily diaries with better reliability were the exception, and objective electronic devices were not used at all. Databank-based investigations generally would provide more objective numbers but could only include falls that led to hospital visits, thus failing to capture the many falls without serious injuries.

Then we had to deal with major disparities in the research design. Several studies used different criteria to define falls: for example, some reported any fall while others required more than one fall, so to allow comparisons between disorders and studies, it would be essential to establish a single definition. Study populations varied greatly in age, disease severity, disease stage, state of self-determination or institutionalization, geographic location, or cultural heritage. The duration of study periods differed widely, ranging from 30 days to one and a half years, making a comparison of incidence rates impossible. To rely on a few studies with large differences in sample sizes limited our power to detect the effects of moderators and publication bias. Finally, in some of the studies, the design was prospective, while in others, it was retrospective or a combination of both. This overall heterogeneity in design and quality, as well as the small number of studies in some of the HKMDs, made it impossible to conduct a meta-analysis of the findings, and our interpretation was limited to a qualitative synthesis. In this respect, it is also important to emphasize that for some HKMDs (e.g., dystonia, tic disorders), no surveys but only case studies have been available, which, although they provide some qualitative insight, cannot be generalized.

Our review has several strengths that need also be highlighted. The search and analysis were done following a highly sensitive search strategy, we used a guideline for discerning viewpoints during the inclusion process, and followed reporting instructions.

Regarding the ramifications of our study, we hope that it will refocus attention on this vastly underappreciated subject with all of its scope and importance and encourage more high-quality research in the area. Furthermore, we are adamant that if more reliable data on the frequency and causes of falls in HKMDs were gathered, it would be feasible to create specific effective prevention strategies that would be able to reduce suffering and expenses significantly. Meanwhile, we believe that our research may make attending physicians and therapists more aware of the higher risk of falling in HKMDs, which may prompt them to inform patients, encourage them to take general safety precautions and to take part in generic preemptive training exercises.

## Conclusion

Thus, despite presenting such a large clinical problem, verifiable and consistent data on fall rates, fall predictors, fall consequences, and fall prevention in HKMD patients are very scarce or not available at all. This highlights the need for further investigations to evaluate falls and related issues in people with HKMDs. We hope this paper will inspire researchers to do more in this field.

However, there is still enough evidence available already to advise the treating physicians. As HKMD patients appear to be a high-risk population for falls, it is important to include a fall history in the routine work-up. In the absence of fall-prevention strategies specific to each of the HKMDs, we would have to derive from those intended for the general public [87] and possibly also apply in part from those for hypokinetic movement disorders like PD [88]. Fall prevention programs may be further tailored as research becomes available to address the specific risks and needs of HKMD patients.

## Additional File

The additional file for this article can be found as follows:

10.5334/tohm.709.s1 Supplementary data. Figure 1. Title: Article identification.
