# Supplementary material for: Accidental Falls in Patients with Hyperkinetic Movement Disorders: A Systematic Review
Source: Tremor Other Hyperkinet Mov (N Y). 2022 Oct 7;12:30. doi: 10.5334/tohm.709 (PMC9541119; doi:10.5334/tohm.709)
Supplement: Supplementary data. — Figure 1. Title: Article identification. [file tohm-12-1-709-s1.pdf]

Supplementary data:

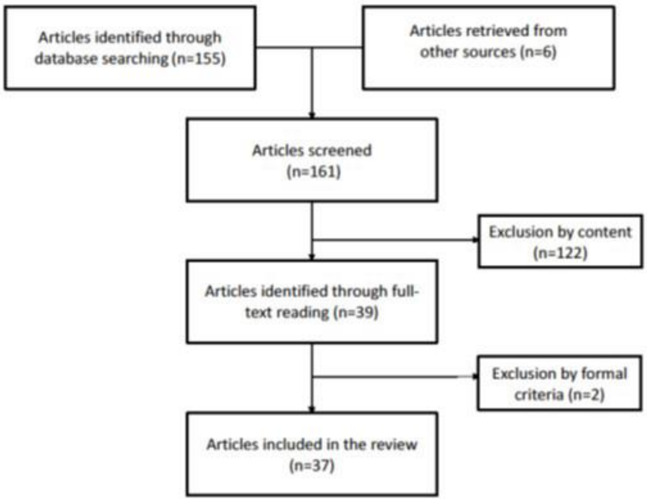

Figure 1. Title: Article identification

Legend: Of 155 articles identified by PubMed searching and 6 articles retrieved through bibliographies, 124 articles were excluded for both content (n=122) and formal reasons (n=2), leaving 37 articles for inclusion in the review.
